# Hybrid Mammogram Classification Using Rough Set and Fuzzy Classifier

**DOI:** 10.1155/2009/680508

**Published:** 2009-10-22

**Authors:** Fadi Abu-Amara, Ikhlas Abdel-Qader

**Affiliations:** Department of Electrical and Computer Engineering, Western Michigan University, MI 49008, USA

## Abstract

We propose a computer aided detection (CAD) system for the detection and classification of suspicious regions in mammographic images. This system combines a dimensionality reduction module (using principal component analysis), a feature extraction module (using independent component analysis), and a feature subset selection module (using rough set model). Rough set model is used to reduce the effect of data inconsistency while a fuzzy classifier is integrated into the system to label subimages into normal or abnormal regions. The experimental results show that this system has an accuracy of 84.03% and a recall percentage of 87.28%.

## 1. Introduction

Breast cancer is the most common cancer among women worldwide. National cancer institute [1] estimates that 192 370-female and 1910-male new cases of breast cancer will appear in the United States in 2009. Also, it is estimated that 40 170 females and 440 males will die of this cancer. Early detection of this disease remains the best known method for reducing its mortality. Also, mammography remains one of the best modalities used by radiologists for early detection of cancerous tumors before clinical symptoms appear. Unfortunately, the growing demand for mammograms is limited by insufficient number of radiologists [2]. A CAD system can be used to assist radiologists in differentiating between normal and suspicious regions, and thus reducing number of unnecessary biopsies and false-positive rates (FP) by the radiologist, FP is an erroneous positive diagnosis when the breast is normal.

Several rough set-based and fuzzy-based methods have been proposed in literature for breast cancer detection. Hassanien and Ali [3] proposed a rough set technique for feature reduction and classification-rule generation from mammographic images. Hu et al. [4] proposed a rough set model (RSM) based on relational algebra that replaces the traditional rough set models. Their proposed algorithm is very efficient in large data sets and may be adaptable for real-time applications. Şahan et al. [5] proposed a hybrid machine learning algorithm by hybridizing k-nearest neighbor algorithm with a fuzzy-artificial immune method where a 10-fold cross validation criterion was used to compute algorithm's accuracy. Hassanien [6] proposed a hybrid method that first uses fuzzy logic to enhance image contrast, extracts region of interest, and enhances its edges. Then, the gray-level cooccurrence matrix is used as a feature extraction method. RSM is used for further subset selection and rule generation and classification. RSM can also be used as a feature selection algorithm [7–10] while fuzzy logic as a classifier [11–13].

In [14], an algorithm was proposed that combined PCA, ICA, and fuzzy classifier for breast cancer detection. Membership functions of fuzzy sets were generated from the product space of the selected features. Also, the selected features from PCA-ICA phase suffered from data inconsistency which degraded the fuzzy classifier performance. In this work, an integration of PCA, ICA, Rough Set, and fuzzy classifier to identify and label suspicious regions from digitized mammograms is developed. Results of this system showed a higher efficiency in detecting suspicious regions and reducing false-negative (FN) rates in comparison with the results of [14] where FN is an erroneous negative diagnosis but the breast tissue has cancer. This work presents a new approach since the mapping range is integrated into the rough set model as opposed to being part of a fuzzy classifier as was the case with [14]. The RSM is integrated into the proposed system as a feature subset selection method in order to reduce the impact of data inconsistency. Finally, the membership functions of the fuzzy sets are based on the mean and standard deviation of the testing data.

In [15] an algorithm was proposed that combined ICA with RSM for breast cancer detection where ICA was used for feature extraction and reduction while in this work PCA is used for feature reduction since PCA is superior to ICA in dimensionality reduction which will enhance the ICA performance, and since it is recommended to preprocess the data through whitening prior to ICA as a tool to reduce the complexity of the problem [16], PCA was a natural choice since whitening is an intrinsic step in PCA.

The novelty of this work is the integration of RSM for feature selection with a fuzzy classifier as well as generating the framework for the integration of the PCA, ICA, RSM, and fuzzy classifier for breast cancer detection. The rest of this paper is organized as follows. Section 2 presents a brief introduction to PCA, ICA, and RSM. Section 3 presents fuzzy logic adaptation while the proposed approach is presented in Section 4. Experimental results are presented in Section 5 followed by Conclusions in Section 6.

## 2. Background

### 2.1. PCA

PCA is an orthogonal transform and a decorrelation technique that captures maximum variance. The correlation between components of a vector is used to measure data redundancy. This means that most of the information contained in the original vector can be represented by a much smaller vector after the PCA stage. In this paper, PCA is used as a dimensionality and noise reduction module. This step ensures that the source components of a vector are uncorrelated.

### 2.2. ICA

ICA is a statistical technique that can be used to extract hidden features within a set of data.

A mammographic image *X* can be expressed as a linear mixture of a set of features or basis functions *a*_*i*_ as shown in (1):

(1) $$x=\underset{i}{\sum }a_{i}s_{i}$$

where *s*_*i*_ are stochastic coefficients that are data dependent. Other transforms such as Wavelets and Gabor assume basis vectors that are independent from data while ICA assumes basis vectors that costumed to the data under consideration. Using matrix notations, (1) can be expressed as shown in (2):

(2) X=AS

where *S* is a matrix contains the source components and *A* is the mixing matrix. This means that a mammographic image consists of a mixture of source components *S*. Their combination can be described using the coefficients of the mixing matrix *A* which can be used as extracted features that describe efficiently any normal and suspicious region.

The ICA algorithm estimates the separating matrix *W* (inverse of *A*) that makes the source components *S* as statistically independent as possible with non-Gaussian (super-or sub-Gaussian) distribution which results in obtaining independent components as shown in (3). This means that *A* should be a square matrix which can be achieved by preprocessing of PCA:

(3) S=WX

The ICA algorithm can be presented as an optimization process of which an objective function is modeled to minimize statistical dependency between the source components. The statistical estimation of the **W** and **S** matrices is a result of this optimization process. The dependency between the source components can be minimized using several suggested methods such as minimizing the mutual information of the components representation [17], maximizing their likelihood [18], or maximizing their non-gaussianity [19, 20].

### 2.3. RSM

Rough set theory can be used as a feature subset selection algorithm. RSM determines and removes the dispensable attributes representing the redundant information within the data while it aims to keep the core attributes representing the minimum essential information.

By relaxing the core algorithm, more attributes can be selected which are called *Reduct*. In this paper, *Reduct* attributes are considered as the minimum selected features. The selected *Reduct* should have the same discernibility and representation power as the original data.

Cardinality is used to replace traditional rough set theory operations. Therefore, algorithm efficiency will be improved with reduced complexity. The cardinality of a set is defined as the number of elements in the set. For example, Table 1 shows three selected features for 8 images (symbols are used instead of pixel values for simplicity). The decision is either normal (*N*_*m*_) or suspicious (*S*) image. The cardinality of Table 1 is

(4) |I|=6  objects,

where *I* = {Feature 1, Feature 2, Feature 3, Decision}. Core attributes should be in every Reduct to ensure correct classification. Therefore, removing any core attribute affects the classifier accuracy. Hu et al. [4] defined the core attributes by (5) as

(5) $$\frac{\left|I-C_{j}\right|}{\left|I-C_{j}-D\right|}\neq 1,$$

where *I* is the decision matrix *I* = [*C*⋮*D*], *C* is the condition attributes (selected features), *D* is the decision attribute (normal or suspicious image), and *C*_*j*_ is the current attribute to be classified as a core or not. The merit value of an attribute or the significance of the attribute is calculated using (6) which is a measure of the degree of dependency for an attribute on the condition and decision attributes:

(6) $$S\left(C_{j}\right)=\frac{\left|I\right|-\left|I-C_{j}\right|}{\left|I\right|}=1-\frac{\left|I-C_{j}\right|}{\left|I\right|}.$$

Two objects are considered consistent if they have the same condition and decision values. For example, in Table 1, the 2nd and the 8th objects are said to be consistent. On the other hand, the 6th and the 7th objects are inconsistent. Inconsistent objects are conflicting objects since they have same selected features but belong to different classes. Rough set model is used in this work to reduce number of inconsistent objects.

## 3. Fuzzy Logic

Human reasoning can be emulated using fuzzy logic. Fuzzy logic is proved to be a powerful tool to handle and process noisy and vague data. Fuzzy rules are more flexible than crisp rules for many reasons. They allow partial set membership and overlapping between fuzzy set definitions which should simplify the classification phase as opposed to crisp rules that are restricted to either a membership or nonmembership to the set. Also, they can be expressed in terms of linguistic statements based on expert knowledge. Finally, the interpretability of the results can be improved by fitting fuzzy rules to the labeled observed data.

**Figure 1:**
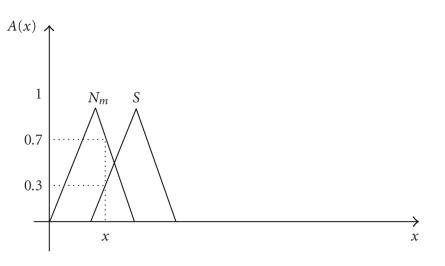
Fuzzy space for an object x consisting of two fuzzy sets: “Normal” and “Suspicious”.

Fuzzy membership functions are easy to implement and they improve speed of inference engines. The difference between normal and suspicious mammographic images may not be well defined. Figure 1 shows, for example, that the object *x* has a membership degree of 0.7 to the fuzzy set “normal” and 0.3 to the fuzzy set “suspicious”.

Several approaches have been developed for automatic derivation of fuzzy rules from the labeled observed data such as genetic algorithm [21], Neuro-fuzzy [22], and fuzzy clustering [23]. In all, the derived fuzzy rules should be accurate, compact, and linguistically interpretable.

Fuzzy if-then rules are used to implement membership function of fuzzy sets as shown in (7):

(7) IF  antecedent  THEN  consequent  [weight].

The weight is a number in the interval [0.0, 1.0] that can be evaluated based on the antecedent numbers. For example, a tested subimage has a membership degree of 0.7 to the fuzzy set “normal” and 0.3 to the fuzzy set “suspicious”. In this case, a single fuzzy if-then rule can be used which produces a classifier output of normal for the tested subimage as shown by the following.

(8) $$\begin{array}{c} \text{If}\;\text{x}\;\text{is}\;\text{normal}\;\left[0.7\right]\text{and}\;\text{x}\;\text{is}\;\text{suspicious}\;\left[0.3\right],\text{then}\\ \text{Y}=\text{normal}. \end{array}$$

Equation (8) is evaluated in two steps. First, a fuzzy operator is applied in order to fuzzify the antecedent numbers. For example, the union fuzzy operator can be applied using (9):

(9) u(a,b)=  max(a,b),

where *a* and *b* are the membership degrees for the membership functions. Applying (9) to the antecedent of (8) will result in selecting the normal fuzzy set from the antecedent with membership degree of 0.7 as follows:

(10) u(a,b)=  max(0.7,0.3)=0.7.

The antecedent results are applied then to the consequent, which is known as the inference step. In this case, the classifier will label the tested subimage as normal.

## 4. Proposed CAD Algorithm

This paper integrates four techniques, namely, PCA, ICA, Rough Set, and Fuzzy classifier to build a CAD system. PCA algorithm is used as a dimensionality and noise reduction tool (prewhitening), and ICA algorithm is used as a feature extraction module while RSM is used as a feature subset selection module followed by a fuzzy classifier.

### 4.1. Data Preprocessing

119 regions of suspicion (ROS) are manually extracted from MIAS database [24] based on center of each abnormality of which 51 are malignant and 68 are benign regions. Two sets are formed where the first set is with subimages of size 45 × 45 while the second set of size 35 × 35 pixels.

**Figure 2:**
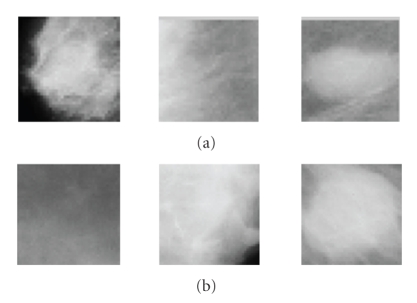
(a) Benign, normal, and malignant subimages of size 35 × 35 pixels and (b) benign, normal, and malignant subimages of size 45 × 45 pixels.

Four other sets of normal subimages are randomly and automatically extracted such that the first set is of size 35 × 35 and the other sets are of size 45 × 45 pixels from the normal MIAS mammograms. Each set has 119 subimages. Each set of ROS is mixed with one set of normal subimages and then divided into two groups: one for the training phase and the other is for the testing phase as shown in Table 2. Figure 2 shows a sample of the extracted subimages.

### 4.2. Training Phase Using PCA-ICA

A training matrix *R*_train_*N*×*M*__ is constructed by placing training subimages as rows in the matrix where *N* represents number of training subimages (119) and *M* represents size of each square subimages. PCA algorithm is used to reduce its dimensionality according to the following equation where *v* represents number of selected principal components and *R*_*M*×*v*_ represents a matrix with the principal components in its columns sorted by descending order according to their variances

(11) $$R_{N\times v}^{R}=R_{{\text{train}}_{N\times M}}R_{M\times v}^{}.$$

In this paper, ICA scheme is based on minimizing the mutual information of the source components which can be achieved using cumulants. This is proposed (a modified version of [17]) in order to estimate the separating matrix *W*and the independent source region matrix *S* in an unsupervised mode as follows.

(i) *W* is initialized to the identity matrix. Then, *S* is calculated using the following equation. This means that ICA is performed on a set of *v* linear combinations of the original subimages instead of performing it on all *N* subimages. This should reduce its computational complexity and hence increase its speed:

(12) $$S_{v\times M}=W_{v\times v}\;{\left(R_{M\times v}\right)}^{T}.$$

(ii) The change in *W* is calculated using the natural gradient [25], that is,

(13) $$\Delta W=\eta \left[I-G\left(S\right)S^{T}\right]\;W,$$

where *η* is the learning rate (step size), *I* is the identity matrix, and *G*(*s*) must be a nonlinear and nonfast growing function. This function is used to measure the statistical dependence between the source components. In this paper, *G*(*s*) [26] is used as follows:

(14) $$G\left(S\right)=f_{1}\left(k_{3},k_{4}\right)\circ S^{2}+f_{2}\left(k_{3},k_{4}\right)\circ S^{3},$$

where *k*_3_ and *k*_4_ are the 3rd and 4th cumulants and (∘) indicates Hadamard product of two matrices and

(15) $$\begin{array}{ll} f_{1}\left(k_{3},k_{4}\right) & =0.5\;k_{3}\left(4.5k_{4}-1\right),\\ f_{2}\left(k_{3},k_{4}\right) & =1.5{\left(k_{3}\right)}^{2}+\frac{1}{6}k_{4}^{}\left(4.5k_{4}-1\right), \end{array}$$

as were defined in [26].

 (iii) The momentum method is used to boost the convergence speed of (13) using

(16) ΔW(t+1)=  ΔW(t)+αΔW(t−1),

where *α* is in the range [0, 1]. In this paper, alpha is chosen to be 0.5.

(iv) The separating matrix is updated and then normalized:

(17) $$\begin{array}{ll} W\left(t+1\right) & =W\left(t\right)+\Delta W\left(t\right),\\ W\left(t+1\right) & =\frac{W\left(t\right)}{\left\|W\left(t\right)\right\|}. \end{array}$$

(v) Stop the algorithm when *W* converges.

Finally, the reduced dimensionality selected features can be estimated as follow.

A minimum square error approximation of the training matrix *R*_train_*N*×*M*__ can be found using the following equation [27] based on (11):

(18) $$\begin{array}{ll} X_{\text{rec}} & =R_{N\times v}^{R}\;R_{M\times v}^{T}\\ & =R_{{\text{train}}_{N\times M}}R_{M\times v}^{}\;R_{M\times v}^{T}\approx R_{{\text{train}}_{N\times M}}. \end{array}$$

From (10),

(19) $$\;{\left(R_{M\times v}^{}\right)}^{T}=W_{v\times v}^{-1}S_{v\times M}.$$

And substitution of (19) into (18) yields

(20) $$X_{\text{rec}}=R_{N\times v}^{R}R_{M\times v}^{T}=R_{N\times v}^{R}W_{v\times v}^{-1}S_{v\times M}.$$

Since *X*_rec_ is an approximation of *R*_train_ and by comparing (20) with (2), the extracted features from the corresponding training set are estimated using (21):

(21) $$Q_{{\text{train}}_{N\times v}}=R_{N\times v}^{R}W_{v\times v}^{-1}.$$

### 4.3. Testing Phase Using PCA-ICA

First, a testing matrix *R*_test_*N*×*M*__ is constructed, where each testing subimage forms a row in the matrix. Second, its rows are normalized by their mean. Third, The regions in *R*_test__*N*×*M*___ are projected on the reduced data from the training procedure using (22):

(22) $$Q_{t_{N\times v}}=R_{{\text{test}}_{N\times M}}\;R_{M\times v}^{}.$$

The reduced dimensionality extracted features from the corresponding testing set are estimated using (23) which is the same principal as (21):

(23) $$Q_{{\text{test}}_{N\times v}}=Q_{t_{N\times v}}W_{v\times v}^{-1}.$$

### 4.4. Mapping into a Limited Range

The estimated matrices *Q*_train_*N*×*v*__ and *Q*_test_*N*×*v*__ contain *N* rows where each row contains *v* selected features from the corresponding subimage. A linear stretching method is used to map them into a limited range of [0, r] using (24):

(24) $$q\left(x,y\right)=\frac{\left(q\left(x,y\right)-\min\left(q\right)\right)\left(r\right)}{\max\left(q\right)-\min\left(q\right)}.$$

### 4.5. Rough Set Model

There are some inconsistent elements (subimages) in the estimated matrices *Q*_train_*N*×*v*__ and *Q*_test_*N*×*v*__. These elements have same selected features but belong to different classes. Rough Set Reduction is used as a subset selection in order to remove features that cause inconsistency and thus improve classification results.

#### 4.5.1. Training Phase

The proposed training framework can be summarized as follows.

1. The consistent elements from the training matrix are removed. The resulting matrix is *Q*_train_*NN*×*v*__, where NN < *N*.
2. Construct the decision matrix, *I*_*NN*×(*v*+1)_ = [*Q*_train_*NN*×*v*__⋮*D*_*NN*×1_], where *Q* contains the condition attributes (selected features from PCA-ICA phase) and *D* is the decision attribute (1: abnormal, 0: normal).
3. Find the Core attributes using the following procedure.
  - Initialize Core vector into *∅*.
  - Check the cardinality for each attribute *C*_*j*_ ∈ *C*; if it satisfies |*I* − *C*_*j*_|/|*I* − *C*_*j*_ − *D*| ≠ 1, then update core vector as Core = [Core⋮*C*_*j*_].
4. Find *Reduct* attributes using the following procedure which is a modified version of [4].
  - Initialize *Reduct* vector: Reduct = Core.
  - Set *Rest* = *I* − *Reduct* and compute the significance of its attributes using:
    (25) $$S\left(C_{j}\right)=\frac{\left|I\right|-\left|I-C_{j}\right|}{\left|I\right|}=1-\frac{\left|I-C_{j}\right|}{\left|I\right|}.$$
  - Let *C*_max_ be the attribute with the largest significance value, update *Reduct* as: *Reduct* = [*Reduct*⋮*C*_max_]
  - Update Rest = I − Reduct.
  - If K ≥ T or the significance values of the remaining attributes are zeros, stop the procedure. Equation (26) means that *Reduct* has inconsistent elements (with ratio of *T*) greater than or equal to that of the decision matrix:
    (26) $$K=\frac{\text{number}\;\text{of}\;\text{inconsistent}\;\text{rows}\;\text{for}\;\left[\text{Reduct}\vdots \text{D}\right]}{\text{number}\;\text{of}\;\text{inconsistent}\;\text{rows}\;\text{for}\;\text{I}}.$$
  - Else, go to step (II).

#### 4.5.2. Testing Phase

In this step, features are selected from the matrix *Q*_test_*N*×*v*__ in the same order they were selected from *Q*_train_*N*×*v*__ during the training phase.

Finally, *Q*_train_*N*×*vv*__ and *Q*_test_*N*×*vv*__ are reconstructed with selected Reduct features while dispensable features are thrown away.

### 4.6. Fuzzy Classifier

Two single fuzzy if-then rules are used to represent the normal and abnormal fuzzy sets. The membership functions of each antecedent fuzzy set are aggregated using the information about the selected feature values of the training subimages.

The proposed fuzzy-based classification algorithm can be summarized as follows:

1. Two activation functions *μ*_as_*N*×1__ and *μ*_ns_*N*×1__ are initialized to 0 where each element of them represents the aggregated membership functions of the selected feature values for the corresponding testing subimage. These parameters are defined as.
  - *μ*_as_*k*×1__ represents the membership degree of the kth testing subimage to the fuzzy set abnormal.
  - *μ*_ns_*k*×1__ represents the membership degree of the kth testing subimage to the fuzzy set normal where 1 ≤ *k* ≤ *N*.
2. Using (27), membership functions of fuzzy sets of the testing subimages are obtained from the mean and standard deviation of their selected features based on the information from the selected feature values of the training subimages:
  (27) $$A_{ij}\left(x_{j}\right)=\exp\;\left(-\frac{{\left(x_{j}-\mu_{j}\right)}^{2}}{{2\left(\sigma_{j}\right)}^{2}}\right),$$
  where *μ*_*j*_ represents mean of all samples of the current selected feature *x*_*j*_, *σ*_*j*_ represents their standard deviation, and *i* is an index for the selected features from the training phase.
3. The membership functions are normalized using
  (28) $$A_{ij}\left(x_{j}\right)=\frac{A_{ij}\left(x_{j}\right)}{\text{ma}{\text{x}}_{ij}\left(A_{j}\left(x_{j}\right)\right)}.$$
4. The membership functions are aggregated using (29) in order to find the degree of activation of each fuzzy set where *i* is an index for the selected features from the testing phase:
  (29) $$\mu_{i}\left(x\right)=\overset{N}{\underset{j=1}{\sum }}A_{ij}\left(x_{j}\right).$$
5. By assigning the corresponding testing subimage into the fuzzy set with the maximum degree of activation, a crisp decision is made, that is, normal or abnormal. Equation (30) is used for this purpose where *C* is used as an index of a testing subimage being identified as normal or abnormal:
  (30) $$C=\max\left(\mu_{\text{as}}\left(x\right),\mu_{\text{ns}}\left(x\right)\right).$$

## 5. Experimnetal Results

Table 3 presents results of using PCA-ICA-Rough-Fuzzy (PIRF), PCA-ICA-Fuzzy (PIF), PCA-Fuzzy (PF), PCA-Rough-Fuzzy (PRF), ICA-Fuzzy (IF), and ICA-Rough-Fuzzy (IRF) in terms of accuracy, recall, precision, FN rates, and FP rates as computer-aided detection systems. Algorithm accuracy is defined as the ratio between the total number of correctly classified subimages to the total number of testing subimages.

Table 4 compares the performance of these CAD systems. Our proposed PIRF CAD system shows a robust performance in comparison with the other algorithms. For example, PIRF achieved an average accuracy of 77.73%, PIF of 75.21%, IRF of 74.16%, PRF of 71.85%, PF of 71.64%, and IF of 49.58%. As Table 3 shows, PIRF has the highest recall percentage among all the other algorithms while it has an average precision of 73.33%. PIF and IRF have average precision of 75.83% each.

As the results show, fuzzy classifier cannot be implemented with ICA model alone without a dimensionality reduction since, without it, a large number of membership functions will be generated. Also, without a feature subset selection module, the classifier task complexity is increased and performance is degraded. Furthermore, results indicate that integrating ICA model with PF generated better results than integrating RSM with PF. The average accuracy was improved by 4.68% and false negative rates were improved by 4.76% if a PCA model was used with the ICA model while following it with RSM improved its average accuracy by 0.29% and its FN rates by 6.33%. Integrating RSM improved total PF algorithm performance by 0.29% but degraded its FN rates by 6.34%. Results also indicate that RSM and PIF integration improves accuracy with an average of 3.35%. 

Comparing the results using FN rates, we find that PIRF has an FN of 8.82%, PIF of 12.61%, IRF of 13.66%, PF of 13.24%, PRF of 14.08%, and IF of 40.34%. Results indicate that using PCA as a dimensionality reduction module reduces FN rates in PIRF and PF at the expense of a little increase in the FP rates. Also, average FN rates are very close to average FP rates in PIF and PRF algorithms. On the other hand, average FN rates are increased in IRF and IF algorithms when no dimensionality reduction was integrated. Finally, integrating RSM into PIF and PF algorithms reduces the number of principal components required to obtain Reduct. The previous discussion shows that each one of the integrated techniques (PCA, ICA, RSM, and Fuzzy Classifier) is necessary and should be implemented in the proposed sequence in order to achieve the highest accuracy rates.

An implementation of the PIF proposed in [14] reports, Table 3, a lower accuracy than our proposed PIRF system in two testing sets while they had same accuracy in the other two testing sets. The average accuracy of the PIF in all test sets is 75.21% while 77.73% for PIRF. FN rates improved in three testing sets for the PIRF in comparison with the PIF. The average FP and FN rates of the PIF are 12.19% and 12.61%, respectively, while 13.45% and 8.82% for PIRF. These observations are summarized in Table 5.

The average accuracy for PIF improved by 3.35% with PIRF system and its average FN rate improved 30.01%. Also, the average selected number of principal components in PIRF algorithm which is 7.75 is less than that of PIF algorithm which is 9.75. In other classification methods such as in [15], three sets of sizes 20 × 20, 40 × 40, and 60 × 60 pixels were extracted from MIAS mammographic images where each set consists of 330 subimages. Their results were 65.71%, 59.36%, and 82.22% for the three sets using ICA-Rough algorithm and 81.9%, 88.57%, and 69.27% using PCA-Rough algorithm.

The proposed CAD system uses several parameters that impact performance accuracy such as number of the principal components in the PCA algorithm, learning rate and alpha in the ICA algorithm, threshold in the Reduct process, and mapping range.

**Number of PCs Selected**

Reducing data dimensionality using PCA module affects PIRF algorithm accuracy. When large number of principal components is selected, extracted features will have redundant information and therefore will degrade the performance accuracy. However, if a small number is selected, extracted features cannot be estimated precisely and the fuzzy classifier performance will also be degraded.

Table 6 shows the highest accuracy for four testing sets using different numbers of the selected principal components while the other parameters are kept constant. Results also show that selecting less than 9 principal components achieves best results in all cases which means that less than 0.65% of the image features are selected for the 35 × 35 subimages and less than 0.4% of the image features are selected for the 45 × 45 subimages. This is in agreement with all reported literature that used PCA algorithm for dimensionality reduction [14, 15].

**Figure 3:**
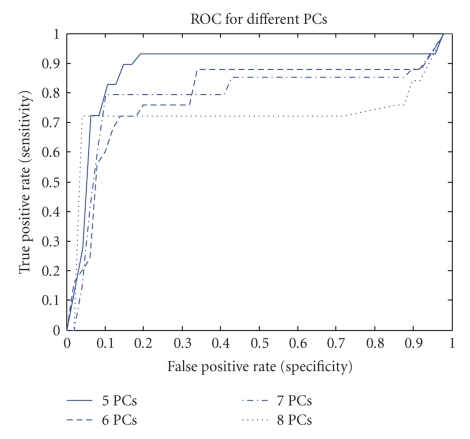
ROC plot for different values of selected principal components for testing set number 4.

On the other hand, Figure 3 shows the Receiver operating characteristic (ROC) plot for a different number of selected principal components for testing set number 4. This figure is generated by plotting true positive rates against false positive rates. As the figure indicates, selecting five principal components produces the largest area under the curve which means that it produces the highest average accuracy.

**Learning Rate**

**Figure 4:**
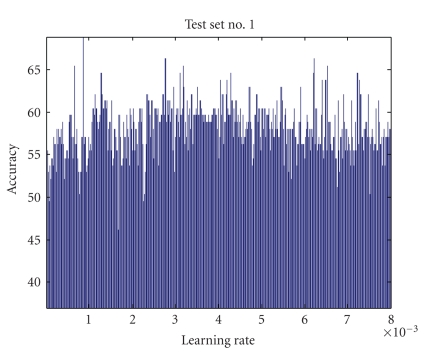
Learning rate impact on accuracy for test set number 1 (all other parameters were kept constant).

**Figure 5:**
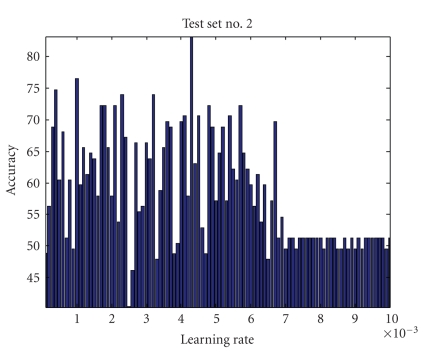
Learning rate impact on accuracy for test set number 2 (all other parameters were kept constant).

**Figure 6:**
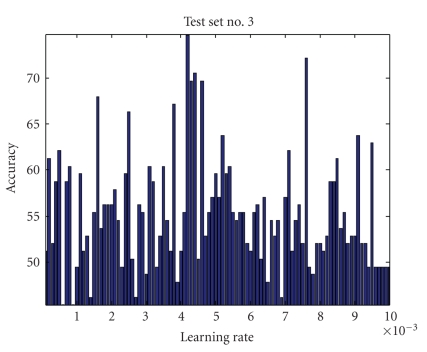
Learning rate impact on accuracy for test set number 3 (all other parameters were kept constant).

**Figure 7:**
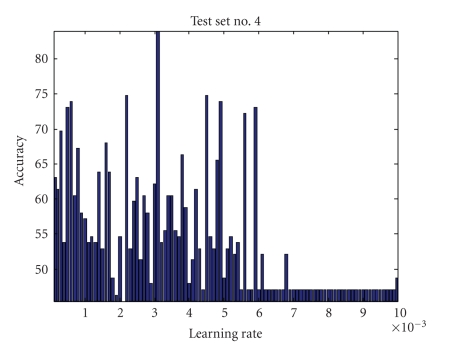
Learning rate impact on accuracy for test set number 4 (all other parameters were kept at constant values).

**Figure 8:**
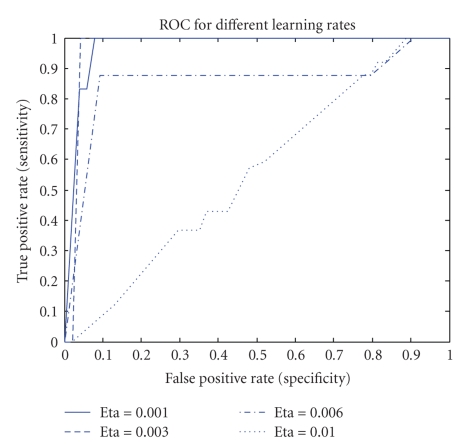
ROC plot for different values of the learning rate for testing set number 4.

The estimation of the matrices *W* and *S* is affected by the learning rate, which determines the speed and accuracy of convergence to the optimal value. Since optimal values of *W* and *S* are unknown and they are data dependent, optimal value of *η* cannot be estimated adaptively. Also, since *η* represents the step size for Δ*W*, choosing a small value of it ensures accuracy but reduces the speed of convergence. Learning rate impact on four testing sets is shown in Figures 4, 5, 6, and 7  where all parameters were kept fixed except for the learning rate. Figure 8 shows the ROC plot for different values of the learning rate for testing set number 4. As the figure indicates, the smallest value of *η* (0.001) produces the largest area under the curve which means that it produces the highest average accuracy.

**Momentum Method Constant**

This constant determines the ratio of the previous Δ*W* that should be added to the current Δ*W* to increase the convergence speed of *W*. Since Δ*W* utilizes the natural gradient to find direction of *W* toward a minimum point, adding its previous value to its current value pushes it toward the minimum point faster but does not change its direction.

**Mapping Range**

**Figure 9:**
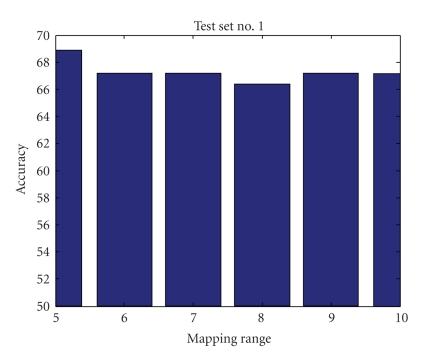
Mapping range impact on accuracy for test set number 1.

**Figure 10:**
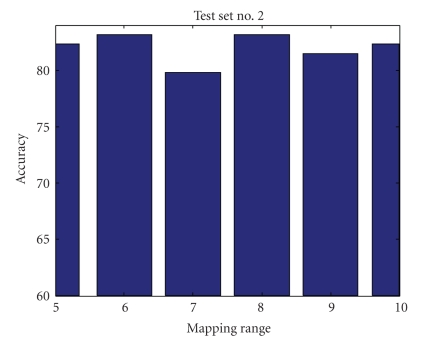
Mapping range impact on accuracy for test set number 2.

**Figure 11:**
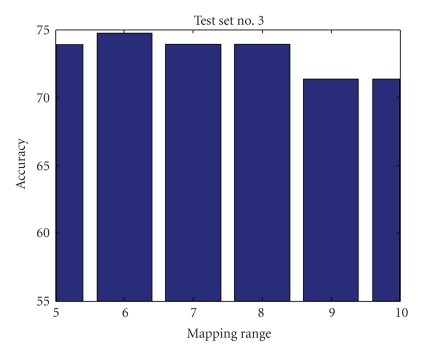
Mapping range impact on accuracy for test set number 3.

**Figure 12:**
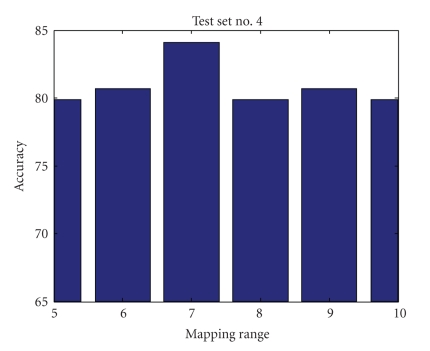
Mapping range impact on accuracy for test set number 4.

**Figure 13:**
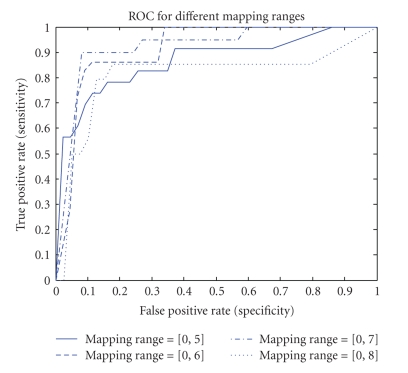
ROC plot for different values of the mapping range for testing set number 4.

In investigating the mapping range values' effect on the accuracy of the results, we found that mapping the data into a limited range results in accuracy loss but simplifies computational complexity and processing time. Figures 9, 10, 11, and 12  show accuracy results versus mapping range for four testing sets while other parameters are kept constant. Figure 13 shows the ROC plot for different values of the mapping range for testing set number 4. As the figure indicates, choosing a mapping range in the interval [0, 7] produces the largest area under the curve which correlates with the highest average accuracy.

**Threshold-*T***

A threshold value *T* is necessary, (26), as a criteria to stop the Reduct procedure. This determines the number of selected features and consequently affects the classifier accuracy. Table 7 shows the impact of *T* on results of test set number 1. These results indicate that selecting a threshold equal to 1 achieves the highest performance. The optimum *T* value is the value, at which the Reduct attributes are complete, at which the number of inconsistent rows equals to that of the decision matrix. Furthermore, the cropped size impacts the accuracy of the results as shown in Table 3. As the table shows, the larger subimages (of size 45 × 45 pixels) resulted in the highest accuracy.

## 6. Conluding Remarks

A computer-aided detection system has been developed and implemented by integrating PCA, ICA, RSM, and a fuzzy classifier. Its performance is compared against the performance of PCA-ICA-Fuzzy, PCA-Fuzzy, PCA-Rough-Fuzzy, ICA-Fuzzy, and ICA-Rough-Fuzzy algorithms.

Results from Tables 3  and 4  indicate that PCA algorithm should be used in order to reduce FN rates at the expense of FP rates. It is shown that integrating RSM and PCA in one algorithm allows for a lower number of principal components to be selected while maintaining the performance accuracy as opposed to use PCA without RSM. Using ICA model and fuzzy classifier produced a CAD system with poor performance unless PCA is used for dimensionality reduction. RSM is used for further features reduction in order to reduce data inconsistency and consequently improve classifier performance. Results also indicate that PCA algorithm should be followed be ICA algorithm instead of RSM. Results of Table 3 indicate that the proposed PIRF algorithm is robust in comparison with the other algorithms. Finally, the proposed CAD algorithm reduces the FN rates considerably which is the main concern of CAD systems.

Parameter values as well as block size play a vital role in the system's performance and an investigation of this relation and perhaps automation of their selection is needed to further improve system's robustness. Although cumulants offer simple computations, they are sensitive to outliers (large values within the set). Therefore, an alternative route that may be worthwhile to investigate is to use a learning rule of the ICA algorithm that is based on negentropy instead of cumulants.

## Figures and Tables

**Table 1:** Selected features for eight images.

Image	Feature 1	Feature 2	Feature 3	Decision
A_1_	A	F	C	*N*_*m*_
A_2_	A	F	D	S
A_3_	E	E	C	*N*_*m*_
A_4_	B	E	C	S
A_5_	B	E	C	S
A_6_	A	E	D	*N*_*m*_
A_7_	A	E	D	S
A_8_	A	F	D	S

**Table 2:** Data sets that used in the evaluation of the proposed algorithm performance.

Set no.	Training set			Testing set			
	ROS	Normal	Total	ROS	Normal	Total	Size-pixels

1	60	59	119	59	60	119	35 × 35
2	60	59	119	59	60	119	45 × 45
3	60	59	119	59	60	119	45 × 45
4	60	59	119	59	60	119	45 × 45

**Table 3:** Results of PCA-ICA-Fuzzy, PCA-ICA-Rough-Fuzzy, PCA-Fuzzy, PCA-Rough-Fuzzy, ICA-Fuzzy, and ICA-Rough-Fuzzy algorithms. NA: not applicable.

Algorithm	Set no.	PC	FP	FN	Accuracy	Precision	Recall
PCA-ICA-Rough-Fuzzy	1	8	21.85%	9.24%	68.91%	56.66%	75.56%
	2	7	9.24%	7.56%	83.19%	**81.67%**	84.49%
	3	8	12.61%	12.61%	74.79%	74.99%	74.99%
	4	8	10.08%	5.88%	**84.03%**	80.01%	** 87.28%**

PCA-ICA-Fuzzy	1	8	16.81%	14.28%	68.91%	66.66%	70.18%
	2	20	10.92%	5.89%	**83.19%**	78.34%	**87.02%**
	3	6	12.61%	21%	66.39%	74.99%	64.29%
	4	5	8.4%	9.25%	82.35%	**83.34%**	81.96%

PCA-Fuzzy	1	20	26.05%	10.08%	63.87%	48.33%	70.74%
	2	5	10.08%	14.29%	75.63%	**80.01%**	73.84%
	3	6	12.61%	21%	66.39%	74.99%	64.29%
	4	5	11.75%	7.58%	**80.67%**	76.7%	**83.61%**

PCA-Rough-Fuzzy	1	16	20.17%	18.49%	61.35%	60%	62.06%
	2	5	9.25%	10.08%	**80.67%**	**81.65%**	**80.33%**
	3	6	16.81%	17.65%	65.55%	66.66%	65.57%
	4	8	10.08%	10.08%	79.83%	80.01%	80.01%

ICA-Fuzzy	1	NA	10.08%	40.34%	49.58%	80.01%	50%
	2	NA	10.08%	40.34%	49.58%	80.01%	50%
	3	NA	10.08%	40.34%	49.58%	80.01%	50%
	4	NA	10.08%	40.34%	49.58%	80.01%	50%

ICA-Rough-Fuzzy	1	NA	15.97%	15.13%	68.91%	68.33%	69.48%
	2	NA	10.08%	9.24%	**80.67%**	80.01%	**81.36%**
	3	NA	14.29%	18.49%	67.22%	71.66%	66.15%
	4	NA	8.4%	11.77%	79.83%	**83.34%**	78.12%

**Table 4:** A Comparison of the different computer-aided detection system results.

Algorithm	Best accuracy	Average accuracy	Average FN	Average FP
PIRF	84.03%	77.73%	8.82%	13.45%
PIF	83.19%	75.21%	12.61%	12.19%
IRF	80.67%	74.16%	13.66%	12.19%
PRF	80.67%	71.85%	14.08%	14.08%
PF	80.67%	71.64%	13.24%	15.12%
IF	49.58%	49.58%	40.34%	10.08%

**Table 5:** Observations of the different developed algorithms.

Algorithm	Observations
PIRF	It has the highest accuracy and recall percentage but not the highest precision
PIF	Needs a feature subset selection module and it has the highest average precision
IRF	Needs a dimensionality and noise reduction module and it has the highest average precision
PRF	Needs a feature extraction module
PF	Needs a feature extraction module and a feature subset selection module
IF	Needs a dimensionality and noise reduction module and a feature subset selection module and it has the lowest accuracy and recall percentage

**Table 6:** The influence of the number of PC on accuracy of the results while learning rate, mapping range, and threshold parameters are kept constants.

PC	Set no. 1	Set no. 2	Set no. 3	Set no. 4
5	62.19%	78.99%	69.75%	80.67%
6	63.03%	81.52%	69.75%	80.67%
7	62.19%	**83.19%**	69.75%	81.52%
8	**68.91%**	78.15%	**74.79%**	**84.03%**
9	68.07%	74.79%	73.95%	78.99%
10	67.23%	75.63%	71.43%	73.95%

**Table 7:** Threshold *T* impact on accuracy for test set number 1 while learning rate, mapping range, and PC are kept constants.

*T*	Set no. 1
1	69%
0.75	65.55%
0.5	63.87%
0.25	63.87%
